# An Usability and Acceptability Study for Immersive Virtual Reality Application in People With Alzheimer’s Disease: A Case Series

**DOI:** 10.1002/alz.093588

**Published:** 2025-01-09

**Authors:** Kübra Nur Menengiç, Cengiz Çelikçi, Zeynep Süzme, İpek Yeldan

**Affiliations:** ^1^ Istanbul Aydin University, Istanbul Turkey; ^2^ Istanbul University‐Cerrahpasa, Istanbul Turkey; ^3^ Kadıköy Municipality Alzheimer’s Disease Center, Istanbul Turkey

## Abstract

**Background:**

Virtual reality (VR), an emerging technology that is becoming increasingly widespread, shows promise as an effective rehabilitation strategy for various diseases. The aim of this study was investigating usability and acceptability of VR in people with Alzheimer’s Disease (AD).

**Method:**

Nine people with early and middle stages of AD were included in the study. The immersive VR application was implemented via the BOBO® VR Z Headset. Selected YouTube VR films, such as relaxing animal and space videos, as well as museum tours, were watched. The VR experience of participants was assessed after a 15 minute application. The usability was evaluated with a survey prepared for this study, including questions evaluating aspects such as realism, comfort, enthusiasm, entertainment, and willingness to participate again. Participants' sickness symptoms were evaluated with the Simulator Sickness Questionnaire, and their moods were evaluated with the Brunel Mood Scale.

**Results:**

All participants found the application realistic and exciting. 4 of participants found the goggles uncomfortable and 2 of them stated that they would not want to apply it again. 4 of the participants experienced symptoms of general discomfort, fatigue, eye discomfort, and difficulty in concentrating.

**Conclusion:**

While the application was found to be feasible and acceptable for some participants, it was not for others. The discomfort, especially in the nose part of the headset, appears to be caused by the design factors of the device. Additionally, some participants experienced sickness symptoms. VR has the potential to serve as an alternative, offering a different and enjoyable approach within rehabilitation practices for certain individuals. However, we are of the opinion that the results of the application show individual differences and the immersive VR may not be suitable for all individuals with AD.

